# Peptide markers of aminoacyl tRNA synthetases facilitate taxa counting in metagenomic data

**DOI:** 10.1186/1471-2164-13-65

**Published:** 2012-02-10

**Authors:** Erez Persi, Uri Weingart, Shiri Freilich, David Horn

**Affiliations:** 1 School of Physics and Astronomy, Tel Aviv University, Tel Aviv 69978, Israel; 2 Sackler Faculty of Medicine and the Blavatnik School of Computer Sciences, Tel Aviv University, Tel Aviv 69978, Israel

## Abstract

**Background:**

Taxa counting is a major problem faced by analysis of metagenomic data. The most popular method relies on analysis of 16S rRNA sequences, but some studies employ also protein based analyses. It would be advantageous to have a method that is applicable directly to short sequences, of the kind extracted from samples in modern metagenomic research. This is achieved by the technique proposed here.

**Results:**

We employ specific peptides, deduced from aminoacyl tRNA synthetases, as markers for the occurrence of single genes in data. Sequences carrying these markers are aligned and compared with each other to provide a lower limit for taxa counts in metagenomic data. The method is compared with 16S rRNA searches on a set of known genomes. The taxa counting problem is analyzed mathematically and a heuristic algorithm is proposed. When applied to genomic contigs of a recent human gut microbiome study, the taxa counting method provides information on numbers of different species and strains. We then apply our method to short read data and demonstrate how it can be calibrated to cope with errors. Comparison to known databases leads to estimates of the percentage of novelties, and the type of phyla involved.

**Conclusions:**

A major advantage of our method is its simplicity: it relies on searching sequences for the occurrence of just 4000 specific peptides belonging to the S61 subgroup of aaRS enzymes. When compared to other methods, it provides additional insight into the taxonomic contents of metagenomic data. Furthermore, it can be directly applied to short read data, avoiding the need for genomic contig reconstruction, and taking into account short reads that are otherwise discarded as singletons. Hence it is very suitable for a fast analysis of next generation sequencing data.

## Background

Estimates of the taxonomy and the number of microbial taxa in metagenomic data often rely on 16S rRNA molecules. Taxonomic assignments can be evaluated by comparing the contigs assembled from the data to known ones, e.g. in the Ribosomal Database Project [1]. However, in view of the taxonomic diversity observed in novel data, it is common to bin the observed 16S rRNA into operational taxonomic units (OTUs) [2], and use the latter for estimating the hierarchy and complexity of the data. The question of whether the 16S method should be the sole classifier of bacterial taxa identities has often been raised in the literature. In order to make sense of genetic and ecological variety, Fraser et al. [3] have argued that this should be only one of many considerations that have to be taken into account. In particular, other genomic information should be used. One of the problems associated with 16S is that there often are multiple copies of this gene in a single genome. Their intragenomic variance leads to difficulties, hence Case et al. [4] suggested they should be replaced by the single-copy gene RpoB, to provide an alternative phylogenetic tree. Alternatively, a full proteomic analysis may be argued to provide an even better basis for phylogenetic studies [5].

The alternative that we propose is based on aminoacyl tRNA synthetases (aaRS). They are known to constitute an essential enzyme super-family, providing fidelity of the translation process of mRNA to proteins in living cells. Their importance to the understanding of evolution has been often emphasized in the literature [6,7]. Here we make use of a particular subgroup of these enzymes, called S61 (single proteins of the EC 6.1.1. classification), that are single-genes, i.e. rarely exists more than once on bacterial genomes [8]. This subgroup contains the EC numbers 6.1.1.x with × equal to 3, 4, 5, 7, 9, 10, 11, 12, 15, 16, 18, 19, 21 and 22. The single-protein property has been verified for all bacteria within Swiss-Prot to an accuracy of 98%.

Having to deal with metagenomic data we employ a technology that identifies reads as belonging to aaRS enzymes by searching for the existence of Specific Peptides (SPs) on such reads [9]. We limit ourselves to SPs of length nine amino-acids and more, ensuring that the probability of their appearance at random is negligible. This list (see Section 6 in the Additional file 1), containing 3,949 peptides, serves as a look-up table, whose elements are searched on all reads or contigs of the metagenome.

We will separately study the cases of short reads and longer genomic data (contigs). Both share the same pre-processing stage, in which we turn all genomic nucleotide sequences into amino-acid strings using the six possible translation modes. We will refer to the latter as Putative Peptides (PPs). The SPs that are observed to occur on the largest numbers of PPs define groups of proteomic reads that form the basis of our calculation. The lower bound on the number of species and strains in the data is obtained by deducing how many different genes could have been responsible for the sets of sequences that have been observed.

There are two major differences between applying our methodology to short reads or to long contigs. In the case of short reads we identify all reads that share a single SP. A single SP of length L ≥ 9 amino acids appears only once in a given gene. Although there may be many SPs belonging to the same EC we have to limit ourselves to only one of them in our counting procedure. This is because two short-sequences that contain two different SPs can be part of a single gene. We then compare all the resulting reads and perform the counting by estimating the minimal number of different strings. Since the reads are short, distinguishing between species belonging to the same genus is impossible. Depending on the length of the short reads, chances are high however for distinguishing between different families, classes and phyla. For the case of long sequences or extensive contigs, we can search for sequences that share several SPs of the same EC. Here we run into the question whether sequences that differ by few amino-acids represent different strains of the same species or different species. We will confront all these issues. It should however be remembered that when we refer to our 'taxa counting' algorithm it may represent effective numbers of families, when applied to short reads, or effective numbers of species and their strains, when applied to long contigs.

In the following we address different aspects of the methodology offered here. In Methods we present a brief review of SPs and establish that SPs of length of 9 amino-acids and more reduce the number of false positives to a bare minimum. We start the Results section with a discussion of the taxa-counting algorithm. We establish the fact that taxonomic acuity depends on the length of the reads being analyzed. The shorter the reads length, the higher is the taxonomic level that can be analyzed. We then define the mathematical problem of taxa-counting, which is equivalent to finding the chromatic number of a graph, a well-known NP-hard problem. We describe our algorithm on a simple example of short read metagenomic data.

We then turn to applying our methodology to long genomic contigs. We start with a study of an artificial metagenome composed of 64 annotated genomes, which we use to compare the 16S methodology with ours, pointing out the problem of distinguishing different strains from different species. We apply our analysis to the contigs published by a recent study of the human gut microbiome (Qin et al., [10]). Qin et al. conclude that the cohort of bacteria in the study of 124 individuals contains 1000 to 1150 prevalent bacterial species, based on non-redundant protein contigs. Applying our analysis to the same set of prevalent proteins we obtain lower bounds of the order of 500 species. Using the set of all contigs, i.e. not just the prevalent set, we estimate numbers of different strains and species to be of the order of 1000. Finally we process and analyze short read data of the same study [10]. We demonstrate how the method works on such data, increasing further the estimates of numbers of species. We show that errors in the data can be monitored and removed to arrive at a stable estimate of taxonomic counts.

The large bacterial diversity often leads to considerable debate on what "species" are and how they should be defined. Our study derives its power from listings of species and strains in Swiss-Prot. Thus the present conventional wisdom, as represented in this data-base, serves as our guidance. Our primary goal is to estimate the number of taxa in a given metagenome prior to taxonomic identification. Providing lower bounds should help to constraint and monitor other methods and techniques with which the method presented here should be integrated. Community composition is our secondary goal. It is estimated both by comparing the experimental data to known databases and by using the taxa-specific peptides (TSPs) methodology [8].

## Results

### Taxa Counting Algorithm

#### Taxonomic Acuity and Read Length

As a preparatory phase of our analysis, we study pairs of bacterial enzymes of the S61 set from the Swiss-Prot data-base. Each enzyme is assigned the taxonomic hierarchy Phylum → Class → Order → Family → Genus → Species. For each level of the hierarchy, we compare pairs of enzymes belonging to different lower levels of the hierarchy. For an identified single pair of proteins we select amino-acid sequences of size W (on a sliding window) from the shorter protein, and count the total number of times that any of these sub-sequences appear in the longer protein without any modification. From this we deduce the probability that a given window had no identical match. Summing over all windows and pairs of enzymes we define the probability of inequality, *P_ine _= N_dif_/Σ*, where *N_dif _*is the number of windows for which no identical match was found, and *Σ *is the number of all windows over all pairs of enzymes compared.

**Figure 1:**
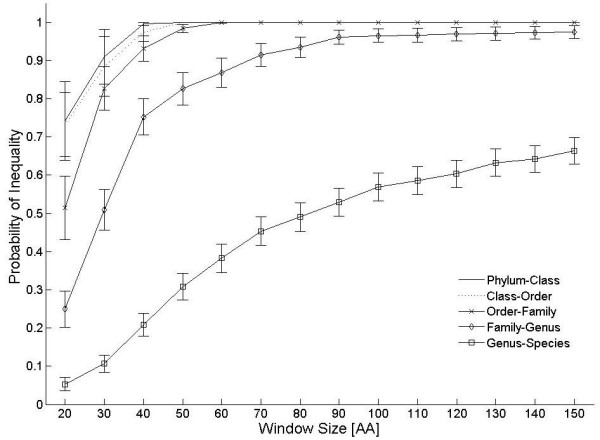
**Probability of inequality *vs *window size**. Probability of inequality *vs *window size, as deduced from S61 enzymes of bacteria in Swiss-Prot. The curves are ordered according to the legend of higher-lower hierarchies, testing for probabilities of inequality of sequences belonging to two different members of the lower hierarchy within the same higher hierarchy.

For each of the W values displayed in Figure 1 we present the probability of inequality, i.e. the probability that a pair of enzymes may be judged, on the basis of windows of length W, to be non-identical amino-acid sequences. These analyses were carried out for any two consecutive taxonomic levels in the hierarchy. We note from Figure 1 that given short reads of 30 amino-acids we can tell apart different families at 80% probability and different genera at 50%, while for 50 amino-acids genera differentiation probability increases to 80% and family distinction is close to 100%. The higher end of the protein length scale is needed to distinguish species and strains, and will be further analyzed below.

#### Taxa Counting Methodology

Let us study a set of amino-acid sequences, all carrying the same SP. We define two sequences *A *and *B *to be *consistent, A~B*, if throughout the domain of their overlap they are identical with one another. Otherwise, i.e. if within their domain of overlap there exists at least one amino-acid that differs between *A *and *B*, they will be designated as *inconsistent, A × B*. Consistent strings may be combined (or fused) into a longer string (unless one fits into the other) that could be part of a single protein. Inconsistent strings belong to different proteins, which imply different taxa because they carry the same SP. The number of inconsistent combinations provides therefore a lower bound on the number of taxa. Throughout this analysis we assume that all sequences are free of errors. We will return to the question of errors later on.

As an example consider the following sequence strings: *A = a-s-b, B = a-s-b-c, C = d-s-b, D = s-b-e. A, B, C *and *D *are the four studied sequences, and *s *represents the SP that is common to them. *a, b, c, d*, and *e *are different sub-strings. Relationships among the sequences are: *A~B, A×C, A~D, B×C, B×D, C~D*. Trying to fuse them into larger strings, we find that the smallest possible (non unique) fulfillment of these constrains is presented by two fused strings: *AB = a-s-b-c, CD = d-s-b-e*. Hence a lower bound of two taxa is expected.

The taxa counting problem, as defined and exemplified above, can be cast into a graphical formulation. Define the graph *G = (V, E) *where the vertices *V *are the sequences and the edges *E *are the inconsistency relations. Thus the example described above can be represented by the graph *A—C—B—D*.

Our problem is equivalent to finding the chromatic number of the graph, i.e. the minimal number of independent groups that cover the whole graph. This is also the minimal number of colors of *V *such that every two connected *Vs *have different colors. In the simple example above the two colors specify *AB *and *CD *respectively. This is a well-known NP-hard problem [11]. Since, however, our problem deals with specific string structures, its solution can be readily estimated even for thousands of reads, as demonstrated below.

To explain our heuristic algorithm with a realistic example we study a set of Rios Mesquites metagenomic data [12]. Using all short read genomic data, converting them to amino-acid strings in six possible ways, and searching for SP hits, we find the following example of 14 short reads exhibiting hits of the SP = FYALPQAPQ, associated with EC = 6.1.1.12 (aspartate-tRNA synthetase), displayed in Table 1. This is the largest number of short reads found to accommodate a single SP of S61 with length ≥ 9.

**Table 1:** Taxa-counting example of Rios Mesquites metagenomic short reads carrying a common SP.

Index	Short read (translated to amino-acid string)	
1	**ILTSSSPEGARDFLVPSRLNPGKFYALPQAPQQFKQLI**	

2	**VFFSFLLGFTKGKFYALPQAPQTILSNLFMVSGFDKYFTNC**	**X**

3	**PSRLNPGKFYALPQAPQQFKQLIMVSGFDRYFQIAPCFR**	

4	**DFLVPSRLHKGKFYALPQAPQQFKQLVMVSGFDKYFQI**	**X**

5	**RFFSSFLGLHKGKFYALPQAPQQFKLTCHGIRVILSN**	**X**

6	**GARDFLVPSRLNPGKFYALPQAPQQFKQLIMVSGFD**	

7	**ARDFLVPSRLNPGKFYALPQAPQQFKQLVMVSGFDRYFQI**	**X**

8	**DFLVPSRLNPGKFYALPQAPQQFKQLIMVSGFDKYFQIA**	

9	**DFLVPSRLHKGKFYALPQAPQQFKQLVMVSGFDKYFQL**	**X**

10	**SRLNPGKFYALPQAPQQFKQLIMVSGFDRYFQIAPCF**	

11	**YFLVPSRLHKGKFYALPQAPQQFKLTCHGIRVILSNC**	**X**

12	**QAGCGLYCSKQIKSWKFYALPQAPQQFKQLIMI**	**X**

13	**LNPGKFYALPQAPQQFKQLIMVSGFDRYFQIAPCFR**	

14	**SFKSRKFYALPQAPQQFKQLIMVSGFDRYFQIAPCFG**	**X**

3 = 3U10U13	**PSRLNPGKFYALPQAPQQFKQLIMVSGFDRYFQIAPCFR**	**X**

1U6U8	**ILTSSSPEGARDFLVPSRLNPGKFYALPQAPQQFKQLIMVSGFDKYFQIA**	**X**

The first 14 rows display the short reads. The two last rows are fused strings of short reads used for solving the minimal chromatic number problem. Entries in the last column indicate 10 independent sequences (inconsistent with each other).

Our method is to construct a set of fused strings of short reads, such that all are inconsistent with each other. Their number constitutes a lower bound on the number of taxa. From Table 1 we note that the following 8 short reads are inconsistent with all others and with each other: 2, 4, 5, 7, 9, 11, 12, 14. Hence they can be recognized as belonging to eight different taxa. Eliminating them we are left with 6 short reads: 1, 3, 6, 8, 10, 13. The relationships among them are displayed in Table 2.

**Table 2:** Consistency and inconsistency relations among the 6 sequences of Table 1 that require further analysis.

	1	3	6	10	13	8
1	~	~	~	~	~	~

3	~	~	~	~	~	X

6	~	~	~	~	~	~

10	~	~	~	~	~	X

13	~	~	~	~	~	X

8	~	X	~	X	X	~

Our heuristic algorithm for solving the problem is based on proposing possible mergers of the short reads into a minimal number of fused strings, proceeding along the following steps:

1) Organize consistency relationships in blocks.

2) Sort short reads by their length in ascending order. (in this case 6 ≥ 10 ≥ 13 ≥ 1 ≥ 3 ≥ 8)

3) For each row, combine short reads with one another and construct fused strings:

a) Within a row, short reads that are substrings of the longest short reads are fused into it. Here, 10, 13 are included in 3, and eliminated from further consideration.

b) Remaining short reads are candidates for possible prolongation of the longest one, creating a new fused string. Here 3 can be prolonged by 1, 6, and 8 can be prolonged by 1, 6 as well. The latter was the arbitrary choice made in Table 1. In general proceed according to size of blocks and size of short read, which in this case were the same.

4) Check if in the resulting list of strings all are inconsistent with all. Otherwise reiterate. Here reiteration is unnecessary; hence we conclude that the smallest number of possible taxa is 8+2 = 10.

The fused string procedure bears similarity to known contig assembly procedures. Nonetheless note that a major difference is that all strings contain the same SP, and no other strings are being used. Moreover, since we will apply the method also to long strings that are contigs to begin with, we keep the term 'fused strings' in order not to confuse them with other assembly procedures.

**Figure 2:**
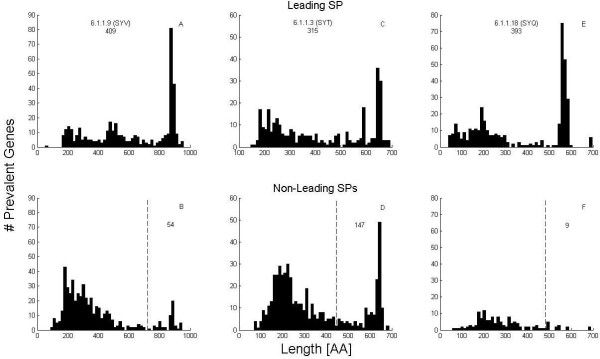
**Length distributions in the prevalent gene set**. Histograms of length distributions of putative proteins of EC = 6.1.1.9, 6.1.1.3 and 6.1.1.18 among the prevalent gene set. Upper figures: sequences carrying the leading SP in all three cases. Lower figures: sequences carrying all other SPs. Vertical dashed lines delineate the range of large sequences. The numbers of the latter are written to the right of the dashed lines. These numbers are added to those of the upper figures in order to form lower bound estimates of taxa counts.

A complete description and Matlab code of the algorithm is available online at: http://horn.tau.ac.il/SC.html. It takes seconds to run on the problem of Table 2. More complicated problems, such as the many long contigs whose results are displayed Figure 2, are completed within a minute on a PC. Existing heuristic algorithms for solving a general chromatic number problem are limited to less than 100 vertices. We have compared our results on tens of contigs in the gut microbiome project (to be discussed below) with those of the chromatic number algorithm on Maple 14 (Waterloo Maple, Inc., Ontario, Canada) and verified that they agree with each other.

### Taxa Counting on Contigs

#### Comparison of the 16S rRNA and S61 SP methods on an artificial metagenome

To elaborate on the differences and similarities between our method and the conventional method based on the analysis of 16S rRNA genes, we have analyzed an artificial metagenome composed of 64 genomes of species and strains (Table 3). Taxa were selected such that they fairly represent the bacterial taxonomic diversity across the tree-of-life [13]. For some of the principal phyla, we have selected pairs of strains of the same species, such that the resolutions of the taxonomic delineation of the two methods can be tested and compared.

**Table 3:** 64 taxa analyzed by 16S rRNA conventional blast analysis and by the new peptide based approach.

Kegg	Taxon Name	Blast 16S	Peptides S61	Specific Peptide
ban	Bacillus anthracis Ames	100%	100%	ISRQLWWGH
			
bar	Bacillus anthracis Ames 0581			ISRQLWWGH

cgb	Corynebacterium glutamicum ATCC 13032 Bielefeld	100%	100%	ISRQLWWGH
			
cgl	Corynebacterium glutamicum ATCC 13032 Kyowa Hakko			ISRQLWWGH

sar	Staphylococcus aureus MRSA252	100%	> 99%	ISRQLWWGH
			
sas	Staphylococcus aureus MSSA476			ISRQLWWGH

sbl	Shewanella baltica OS155	100%	> 99%	ISRQLWWGH
			
sbm	Shewanella baltica OS185			ISRQLWWGH

ypa	Yersinia pestis Antiqua	100%	100%	ISRQLWWGH
			
ypg	Yersinia pestis Angola			ISRQLWWGH
			
ypp	Yersinia pestis Pestoides			ISRQLWWGH

bfr	Bacteroides fragilis YCH46	> 99%	100%	ISRQLWWGH
			
bfs	Bacteroides fragilis NCTC9343			ISRQLWWGH

cbf	Clostridium botulinum F	> 99%	100%	ISRQLWWGH
			
cbo	Clostridium botulinum A			ISRQLWWGH

cpa	Chlamydophila pneumoniae AR39	> 99%	100%	ISRQLWWGH
			
cpj	Chlamydophila pneumoniae J138			ISRQLWWGH

cta	Chlamydia trachomatis serovar A	> 99%	100%	ISRQLWWGH
			
ctr	Chlamydia trachomatis serovar D			ISRQLWWGH

eci	Escherichia coli UTI89 UPEC	> 99%	> 99%	ISRQLWWGH
			
eco	Escherichia coli K-12 MG1655			ISRQLWWGH

llc	Lactococcus lactis subsp cremoris SK11	> 99%	> 99%	ISRQLWWGH
			
llm	Lactococcus lactis subsp cremoris MG1363			ISRQLWWGH

mtc	Mycobacterium tuberculosis CDC1551	> 99%	100%	ISRQLWWGH
			
mtf	Mycobacterium tuberculosis F11			ISRQLWWGH

sag	Streptococcus agalactiae 2603 serotype V	> 99%	> 99%	ISRQLWWGH
			
san	Streptococcus agalactiae NEM316 serotype III			ISRQLWWGH

stc	Streptococcus thermophilus CNRZ1066	> 99%	> 99%	ISRQLWWGH
			
ste	Streptococcus thermophilus LMD-9			ISRQLWWGH

syd	Synechococcus sp CC9605	> 99%	V	ISRQLWWGH
		
sye	Synechococcus sp CC9902		V	ISRQLWWGH

bbr	Bordetella bronchiseptica	> 99%	V	ISRQLWWGH
		
bpe	Bordetella pertussis		V	ISRQLWWGH

pmf	Prochlorococcus marinus MIT 9303	V	V	ISRQLWWGH

pmh	Prochlorococcus marinus MIT 9215	V	V	ISRQLWWGH

tle	Thermotoga lettingae	V	V	ISRQLWWGH

tma	Thermotoga maritime	V	V	ISRQLWWGH

bth	Bacteroides thetaiotaomicron	V	V	ISRQLWWGH

cau	Chloroflexus aurantiacus	V	V	ISRQLWWGH

cdi	Corynebacterium diphtheria	V	V	ISRQLWWGH

cef	Corynebacterium efficiens	V	V	ISRQLWWGH

cha	Campylobacter hominis ATCC BAA-381	V	V	ISRQLWWGH

cje	Campylobacter jejuni NCTC11168	V	V	ISRQLWWGH

cmu	Chlamydia muridarum	V	V	ISRQLWWGH

cph	Chlorobium phaeobacteroides	V	V	ISRQLWWGH

det	Dehalococcoides ethenogenes	V	V	ISRQLWWGH

dge	Deinococcus geothermalis	V	V	ISRQLWWGH

dra	Deinococcus radiodurans	V	V	ISRQLWWGH

gfo	Gramella forsetii	V	V	ISRQLWWGH

rpd	Rhodopseudomonas palustris BisB5	V	V	ISRQLWWGH

aav	Acidovorax avenae	V	V	ISRQLWWGH

abu	Arcobacter butzleri	V	V	ISRQLWWGH

ade	Anaeromyxobacter dehalogenans	V	V	ISRQLWWGH

atu	Agrobacterium tumefaciens C58 UWashDupont	V	V	ISRQLWWGH

rpa	Rhodopseudomonas palustris CGA009	V	V	ISRQLWWGH

aae	Aquifex aeolicus	V	V	FFWVARMIM

cch	Chlorobium chlorochromatii	V	V	FFWVARMIM

chu	Cytophaga hutchinsonii	V	V	FFWVARMIM

fnu	Fusobacterium nucleatum	V	V	FFWVARMIM

rba	Rhodopirellula baltica	V	V	DTWFSSALWP

gme	Geobacter metallireducens	V	V	DTWFSSALWP

aau	Arthrobacter aurescens	V	V	DDNGLPTER

mga	Mycoplasma gallisepticum	V	V	DTWFSSALWP

mpe	Mycoplasma penetrans	V	V	ISRQLWWGH

Kegg code and taxa names are given in the first 2 columns. Last three columns display the results of the two methods. **Blast 16S analysis (3rd column)**: strains of the same species that were both fully matched (100%) to the same 16S rRNA query hence cannot be distinguished. Additional taxa that cannot be distinguished at an identity threshold of 99% are also indicated. 'V' represents taxa distinguished from each other with an identity level > 99%. **S61 Peptide analysis (4th column)**: strains of the same species that were fully matched, i.e. no difference between the corresponding two sequences exist within the tested range (100%). Additional strains that cannot be distinguished if up to 4 amino-acid differences are allowed between the sequences (99%). 'V' represents taxa distinguished from each other with an identity level > 99%. **Last column**: SP identification of the sequence.

Full DNA sequences of all taxa were analyzed by the two methods. Following the conventional method we have retrieved the full length 16S rRNA sequences for all 64 selected taxa (from the Kyoto Encyclopedia of Genes and Genomes (KEGG)). BLAST search was then used to map the 16S rRNA genes to the artificial database which contained the complete DNA cohort of these 64 taxa. We have analyzed the results after initial filtering by requiring identity match > 97% and 80% alignment overlap, considering the length of the query sequence. For each taxon we identified at least one 16S rRNA copy that was fully matched to the right DNA sequence (100% identity blast match). However, in several cases a 16S rRNA sequence has a match with more than a single taxon. We found 5 species whose strains (4 doublets; 1 triplet) match by 100% to the 16S rRNA gene copies. For taxa-counting this means that 6 strains cannot be distinguished, hence a count based on 16S will lead to 58 species (and strains). These numbers change if one allows identity match of > 99%. Then, there are additional 11 species whose strains cannot be distinguished, leading to taxa-count of 47. Already at this level of identity > 99% there are 2 different species of the same genus that cannot be distinguished from each other (Table 3 bottom, Bordetella bronchiseptica and Bordetella pertussis). The situation is worse for a cut-off of 97%, when more species cannot be distinguished from each other (e.g., the following species match: Prochlorococcus marinus MIT 9303, Prochlorococcus marinus MIT. 9215, Synechococcus sp. CC9902, Synechococcus sp. CC9605). This is a well-known problem of 16S analysis [3,4,13].

We have performed an analysis by S61 SP search and alignment on the same metagenomic data. The most abundant SP (ISRQLWWGH of EC = 6.1.1.9) was found in 56 out of the 64 genomes (Table 3, last column). The other 8 genomes can be identified through occurrences of other SPs belonging to the same EC. All sequences, including the minority of 8 that do not possess the leading SP, can be easily aligned with each other, exhibiting similarities and differences. Using this procedure, there are several cases where strains cannot be distinguished. They are exhibited in Table 3 (4^th ^column, 100%), where we find 7 strain pairs and 1 strain triplet that fully match one another, i.e. the distances between the corresponding sequences are zero. Analogously to the 99% identity threshold in the 16S analysis, we can set a similar threshold in the enzyme (~ 4 amino-acids difference, given the typical length of these genes), finding 6 additional pairs of strains cannot be distinguished (Table 3, 4^th ^column, 99%).

We will return to the issue of species *vs *strain counting below, after analyzing the metagenomic results of Qin et al., [10]. One important point of caution is that the results may depend on the type of protein that underlies our analysis. Thus for the 6.1.1.9 case, studied here, differences of a few amino-acids in the protein sequence occur for different strains of the same species as well as for different species of the same genus. This is not the case for 6.1.1.18 for example, where distances < 3 amino-acids usually signify different strains of the same species, while larger distances are characteristic of different species. At the resolution level of strains we see that even for an artificial metagenome where 16S rRNA genes are available, the S61 peptide approach can serve as a comparable tool for distinguishing strains from species. It can further serve as a complementary tool to distinguish between strains and species based on the analysis of certain ECs, such as the 6.1.1.18 (see next section). It may be of high importance for environmental samples of unknown taxa as it does not require a predefined query dataset.

Finally we wish to point out that if full genes (or proteins) are being used for analysis purposes, we do not have to confine ourselves to SPs with lengths ≥ 9. Instead we can use a larger list of SPs with length ≥ 7 and look for all cases where sufficient amino-acids (e.g. 9 or more) are hit by these SPs on the protein. This way we may uncover a relevant protein, having the same EC, even if no SP with length 9 or more is observed on it. It should be realized that all examples quoted in Table 3 have many hits of SPs with length 7 or 8, in addition to those mentioned in the last column. For example, the *M penetrans *enzyme (last row) has 15 (partially overlapping) such hits in addition to the leading SP of length 9. Thus, even if the latter would have been corrupted by some mutation, the protein would be identified as EC = 6.1.1.9 by the DME methodology [14] that takes into account all these hits, as demonstrated by the webtool http://adios.tau.ac.il/DME11.html. Our previous limitation to length ≥ 9 was important in the short read application, where two hits of SPs of length 7 on the same read are very rare, and a single SP hit with length 7 may be erroneous (see Methods).

#### Human gut microbial species counting on long genomic contigs - the prevalent set of Qin et al. [10]

The human gut microbial metagenome has recently been studied on samples taken from 124 human individuals [10] and shown to be due to a cohort containing over 1000 prevalent species. This conclusion was based on estimating the contents of a non-redundant set of 3.3 M ORFs derived from full genomic analysis (see Methods). Searching for S61 SPs on the set of all prevalent genes we find that the largest number of sequences recognized by SPs of lengths ≥ 9 occurs for EC = 6.1.1.9. For comparison we will also quote results from ECs 6.1.1.3 and 6.1.1.18. The numbers of sequences with hits of the leading SPs of these three ECs are 409, 315, and 393 correspondingly (Figure 2, upper panel). Summing up all sequences on which non-leading SPs corresponding to these ECs were found (Figure 2, lower panel) we obtain total counts of 912, 839 and 530 correspondingly. It should be noted that, since this is a non-redundant set to begin with, we do not have to apply the taxa-counting algorithm. Moreover, given the stringent definition of prevalent genes (see Methods) we can eliminate the possibility of counting different strains of the same species. The results from ECs 6.1.1.9 and 6.1.1.3 seemingly agree with the species count estimated by the authors [10]. However, there is a flaw in this argument: many of the prevalent gene sequences are only partial sections of the relevant genes. Thus, adding up numbers due to different SP markers, one runs the risk of over-counting, by regarding possible non-overlapping sections of a single gene as putative independent genes.

In Figure 2 (left) we display the length histograms of sequences carrying the leading SP of 6.1.1.9 (ISRQLWWGH, the same as in Table 3) and of all non-leading SPs with lengths ≥ 9. The peak at length of about 900 amino-acids signifies sequences that are full representations of the enzyme SYV (Valyl tRNA synthetase) carrying this EC (see discussion in Additional file 1, Fig. S1). Obviously this peak accounts for only a fraction of the total counts mentioned above. In order to generate a strict lower-bound estimate, we start with the count (409) of the leading SP. Since the latter can only appear once on this enzyme we can regard all the sequences in which it was found as different. However, to add to this count the other sequences of the non-leading SPs, we must consider only sufficiently long sequences, reducing the probability that they are complements of the gene sequences that were already counted. Thus, we add the peak of the non-leading distribution (counting sequences having lengths of 700 amino-acids or more that are known to lack the leading SP, Figure 2B). This way we are guaranteed that no over-counting occurs. This procedure leads to the lower-bound estimate of 463 prevalent species, characterized by one SYV for each species.

Thus whereas 912 different species could lead to the observed 912 different sequences, a rigorous lower bound on prevalent species should be set at 463 on the basis of the available SYV data. The lower bound estimates for all three ECs, are 463, 462 and 402 for the SYV (Figure 2, left), SYT (Figure 2, middle) and SYQ (Figure 2, right) genes correspondingly. Note that while most SYV sequences carry the leading SP, the SYT count relies heavily on non-leading SPs; nonetheless the total numbers are amazingly consistent with one another. SYQ leads to a lower count, reflecting presumably the absence of such genes on some bacteria. This discrepancy is present and even bigger in Swiss-Prot data, as shown in the Additional file 1 (Fig. S2).

#### Human gut microbial taxa counting on all long genomic contigs of Qin et al. [10]

Next we apply our S61 SP analysis to the full set of (redundant) contigs in Qin et al. [10]. By regarding every possible difference among protein sequences as meaningful, we will reach lower bounds on taxa counts (both species and strains) that are much higher than in the previous section. Expanding our algorithm to incorporate different levels of sequence inequality we will demonstrate their consistency with the results on the prevalent set.

We start by translating each contig into six pseudo-peptides (PPs), according to the six different choices of reading frames. SPs are then searched on all PPs of all human microbial populations. SP hits with length of L ≥ 9 amino-acids occur only once on a PP. Combining all relevant PPs of the whole cohort, we search for SPs that lead to the highest numbers of hits. Concentrating on the same ECs that we have investigated in the prevalent set, we find the following hits of leading SPs:

ISRQLWWGH (EC = 6.1.1.9, gene = SYV) 1488 hits.

TRFPPEPNGYLH (EC = 6.1.1.18, gene = SYQ) 1961 hits.

GEAAFYGPK (EC = 6.1.1.3, gene = SYT) 1488 hits.

**Figure 3:**
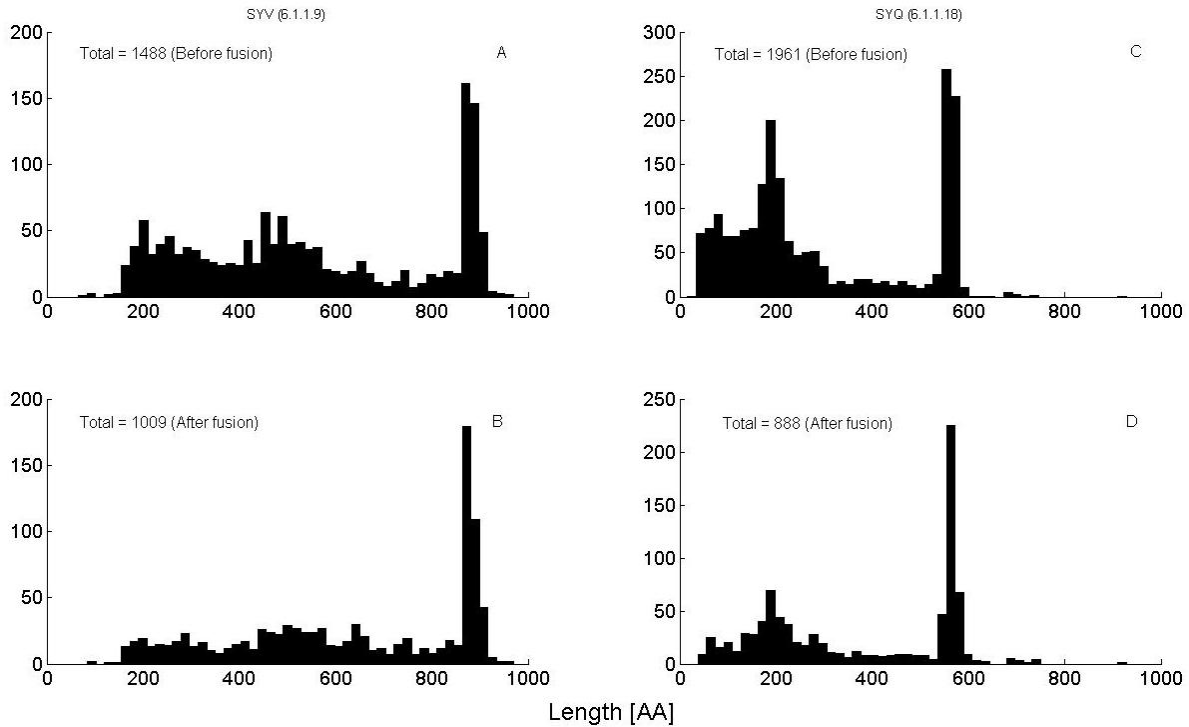
**Length distributions of putative proteins**. Histogram of the length of sections of enzymes. Lengths derived from the 1488 PPs that contain a hit of SP = ISRQLWWGH (EC = 6.1.1.9) before (A) and after (B) obtaining a solution of the minimal number of mutually inconsistent sequences by the taxa-counting algorithm. Lengths derived from the 1961 PPs that contain a hit of SP = TRFPPEPNGYLH (EC = 6.1.1.18), before (C) and after (D) the algorithm.

We extract from all PPs the sections that correspond to the proper enzymatic gene, i.e. start with the first Methionine following the closest Stop signal to the left of the SP, and end at the first Stop signal downstream to the SP. The length histogram of these PPs is presented in Figure 3 for the first (Figure 3A) and second SP (Figure 3C). Most of the proper full enzymatic proteins establish the peaks around contig lengths of 900 and 600 amino-acids for SYV and SYQ respectively. The contigs leading to shorter PPs either begin too late or end too early, thus containing only fractions of an enzyme (see also discussion of SYV data in Additional file 1).

Next we employ the taxa counting algorithm with which we analyzed the examples of Tables 1, 2 to create the assemblies of PPs (Figure 3B, D). In fact, there are many cases where short PPs are completely fused into longer ones. This process converges quickly, within one minute on a regular PC, leading to a lower bound of 1009 and 888 taxa, respectively. Analogous analysis based on the 1488 hits of SP = GEAAFYGPK (EC = 6.1.1.3) leads to a count of 718.

Now we proceed to look for hits of other SPs (of length ≥ 9) of the same EC numbers, on contigs on which the leading SP was not found, to select further candidates for the corresponding enzymes. We limit ourselves to PPs which are long enough so that the most abundant SP was not missed just because the PP does not contain its appropriate location. Thus, when applying the algorithm to the non-leading SPs we exclude fractions of proteins that are shorter than the cutoff lengths that we have employed in Figure 2 (700 amino-acids for SYV, 500 for SYQ and 450 for SYT). This leads to a final total count of 1136 for SYV (EC = 6.1.1.9), 937 for SYQ (EC = 6.1.1.18) and 1076 for SYT (EC = 6.1.1.3). In the next section we will make contact between these numbers and the prevalent gene counts discussed in the previous section.

#### Taxa counting based on minimal distance *d*: differentiating between strains and species

We have demonstrated in Table 3 the problematics of distinguishing between strains of the same species using the 16S rRNA technology as well as ours. There we have employed SPs belonging to 6.1.1.9. Here we wish to point out that the situation changes if one employs 6.1.1.18 which, we have already seen, has quite a large number of species count in this data, although smaller than that of 6.1.1.9.

**Figure 4:**
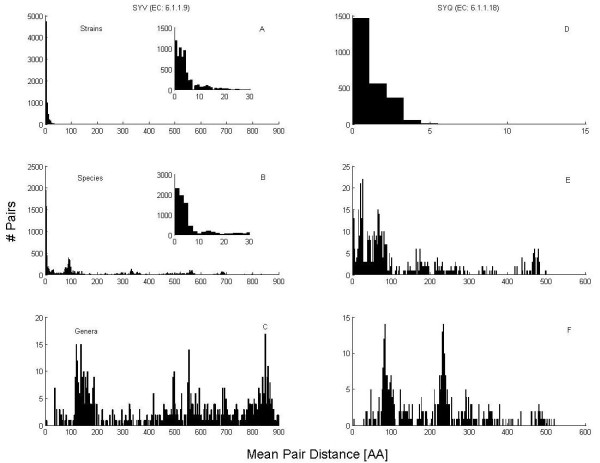
**Hamming distances among enzymes in Uniprot data**. Statistics of Hamming distances between 6.1.1.9 (A-C) sequences and 6.1.1.18 sequences (D-F) in Uniprot KB Data. Top: Differences between strains of the same species (A, D). Middle: Differences between species of the same genus (B, E). Bottom: differences between genera in the same family (C, F). Insets in (A, B) display results for low distances and demonstrate the lack of clear cut-off between species and strains for 6.1.1.9 in contrast with the clear cut-off at distance 2 for 6.1.1.18 enzymes.

In Figure 4 we present the results of a study of all bacterial protein sequences that contain the most abundant SPs for SYV (6.1.1.9) and SYQ (6.1.1.18) in Uniprot KB data. Here we display histograms of pairs of strains in same species, and pairs of species in the same genus, as function of Hamming distance, i.e. differences in amino-acid identities in protein sequences. For completion we also add pairs of different genera in the same family. In order to compare different sequences we aligned them according to their common SP and measured their Hamming distances on overlapping domains. For SYV enzymes, displayed in Figure 4(A-C), we find very similar behavior for both strains and species: both have distributions ranging mostly over differences in amino-acids from 0 to 4. This is in agreement with our conclusions in Table 3, where we saw that it is impossible to set a clear threshold on amino-acid distance below which one finds only different strains of same species. Analyzing the SYQ data in Figure 4(D-F) a clear threshold distinguishing different strains from different species is observed: more than 95% of all strains of the same species differ only by 0-2 amino-acids, whereas about the same majority of different species differ from each other by more than 2 amino-acids. The same consequences hold also for an analysis based on the Needleman-Wunsch edit distance [15], displayed in Additional file 1 Fig. S4.

In order to apply such a threshold criterion to our data we return to the ensembles of fused strings, discussed in the previous section, and reevaluate them according to the minimal difference in amino-acids (Hamming distance) detected among pairs of fused strings. To this effect we construct a distance matrix *M*, whose upper triangle entries signify distances between shorter to longer strings, i.e. the indices of *M *are ordered (from top to bottom and from left to right) according to the length of the string that is being considered.

In order to estimate the minimal number of fused strings whose distance from one another is ≤ *d *we follow three steps:

a) Identify indices of all sequences that belong to a pair having distance ≤ *d*. This leads to an *Nx2 *matrix in which, for each row, the index value in the left column is always smaller than in the right column. *N *is the number of pairs of sequences with distance ≤ *d*.

b) Identify the unique values in each column of the *Nx2 *matrix (i.e. each separate index value is counted only once). The smaller set (usually the left one) of unique values is regarded as the minimal set.

c) Remove from the set of sequences all those corresponding to the minimal set of indices. All remaining sequences have distances >*d*.

**Figure 5:**
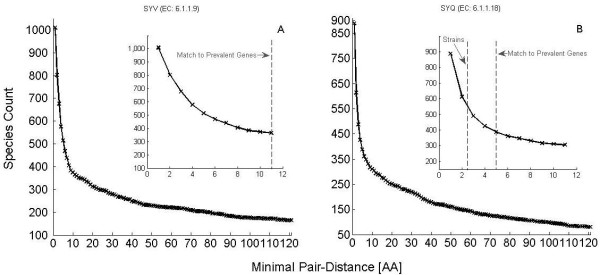
**Taxa counting *vs *minimal distance**. Taxa counting for leading SPs as function of minimal distance *d *for (A) SYV 6.1.1.9 and (B) SYQ 6.1.1.18 for the fused strings that are full protein candidates. Based on the statistics of Swiss-Prot displayed in Fig. 4, we estimate from (B) that 400 of the total count may be due to different strains, exhibiting distance ≤ 2. In comparing with Fig. 4 note that the latter starts with a bin of zero difference between sequences, whereas here the first bin refers to differences larger or equal to 1.

Figure 5 displays the results of applying this procedure to estimate the numbers of fused strings as function of the minimal distances between them. This figure allows us to make contact with the analysis of prevalent genes made in the previous section. The numbers of our prevalent species estimates coincide with the curves of Figure 5 at distances of 11 amino-acids for SYV sequences and 5 amino-acids for SYQ ones. Clearly these species counts do not involve any significant contribution from different strains of the same species.

The SYQ curve allows us to estimate the numbers of different strains of the same species, by using the cutoff of *d *≤ 2 derived from Figure 4D. We learn from it that out of the overall number of 937 taxa derived from SYQ sequences, more than 400 may be associated with different strains of the same species (Figure 5B). Note that many more strains may be indistinguishable on the basis of SYQ sequences, as demonstrated in the Swiss-Prot statistics displayed by the peak at zero distance in Figure 4D. A different way of making the statement regarding the number of strains is that the SYQ analysis implies that there exist more than 490 genuinely different species (Figure 5B), whereas our prevalent species estimate for this EC was 402 only.

Errors in contig constructions affect the counting procedure, since the latter interprets any mutation as a new taxon. Qin *et al. *[10] estimated their assembly errors to be approximately 14 per Mb. From this we deduce that the maximum error we should expect is about 42 wrong amino-acids in an ensemble of PPs containing Million amino-acids. Hence we estimate that our total counts are subject to a possible error which is of the same order of magnitude as the drop encountered in Figure 5 between the peak at *d *≥ 1 and the next point at *d *≥ 2. This implies that the estimate of the number of strains may be subjected to a large uncertainty, however the estimate of numbers of different taxa derived from *d *≥ 2 is quite a safe lower bound.

#### Taxonomic identification - contigs

Both distributions displayed in Figure 5 show a sharp drop for low *d*, turning into linear descent for higher *d *values. No asymptotic plateau is observed at larger *d*, as would be expected if the data had large numbers of genera in it (see Figure 4). Hence we conclude that the number of different genera is relatively small, presumably less than a few tens.

So far our analysis has been carried out without any reference to the taxonomic identification of the species involved. An SP-based approach that can provide an answer to this question has been developed in [8], where Taxa Specific Peptides (TSPs) were filtered out of all SP lists. The TSPs are specific to a phylum or class, and may provide taxonomic classification even on short read data (see below). This method can be easily applied to the contigs of 6.1.1.9. We find taxonomic TSP identification for 471 out of the 1009 fused strings of *d *≥ 1. They contain 220 Bacterioidetes, 168 Firmicutes, 38 classified generally as Proteobacteria, additional 41 classified as Gammaproteobacteria, and a small minority of 4 Tenericutes. Obviously one can also perform a BLAST analysis, e.g. with respect to Uniprot data. It implies that most bacterial species are limited to just two classes, Bacteroidetes and Clostridia, which is consistent with the observation of [10] and others [16], as well as our TSP estimates, that Bacteoidetes and Firmicutes are the dominant phyla in gut microbiomes.

Note that we based most of our analysis on analogies with Uniprot KB data. Its statistics is further discussed in the Additional file 1. The Venn diagram of Additional file 1, Figure S2, shows that most of the recorded species (and their different strains) have both 6.1.1.3 and 6.1.1.9 annotated enzymes, but 2/3 of them lack 6.1.1.18. The distribution observed in the microbiome analyzed here is different, presumably because it is composed of taxa belonging mostly to just Bacteroidetes and Clostridia.

### Taxa Counting on Short Reads

In the previous sections we have studied taxa counting on long contigs. Applying our algorithm to well separated contigs, like the 'prevalent genes' set of [10], we are quite sure to obtain a lower limit on the number of species that are responsible for the observed data. On the other hand, if we study redundant contigs we can obtain some indication on the number of strains involved in these data, by relying on the 6.1.1.18 analysis. Here we wish to extend our study to raw short reads. A large fraction of the latter are often discarded in metagenomic studies because they are classified as singletons, i.e. they cannot be combined with other short reads to form contigs. Our taxa counting can however be applied to all of them. This provides an insight into the data that has been discarded by others. It can further elaborate on community composition.

#### Analysis of human 12 short read data [10]

Qin et al [10] have applied particular deep sequencing searches to two of the human samples, 6 and 12. We have made use of the available Illumina short read data of human 6 and human 12. We performed SP searches after translating each one of the short reads into the 6 possible reading frames. We then looked for PPs in which the leading SPs of SYV (6.1.1.9) and SYQ (6.1.1.18) were found. For human 12 we found sets of 1780 SYV sequences and 1485 SYQ ones.

**Figure 6:**
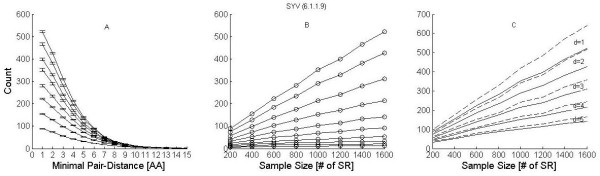
**Taxa counting from short read data**. Taxa count analysis for SYV (EC: 6.1.1.9) based on the 1780 Illumina short reads sharing the common leading SP. A) Counts are displayed as function of the minimal Hamming distance between fused strings. Different curves represent different sample sizes varying (from bottom to top) from S = 200 to 1600. Mean values and errors on the mean are calculated from 20 random realizations at each sample size. B) Mean counts as function of sample size S, for *d ≥ 1-10 *(top to bottom). C) Mean counts as function of sample size for *d ≥ 1-5 *for the data in B (solid) and for artificial data (dashed) constructed from the real data into which artificial errors were introduced with probability of 1% per amino acid.

Based on these sequences, we demonstrate what consequences may be drawn with regards to taxa counts and taxa composition. On the basis of these data we test, as well, the variation of these results with respect to depth of analysis, i.e. the number of sequences that are being taken into consideration by the taxa counting algorithm. For this purpose we choose random sets of varying sample sizes *S < N_p_*, where *N_p _*is the number of PPs with the common leading SP. For each sample size, *S*, 20 random trials are performed in order to draw conclusions about mean counts and their errors. The results are displayed in Figure 6 for human 12. The left panel displays mean counts and their errors for different sample sizes as function of minimal pair distances *d*. The middle panel displays the same results as function of sample size. Whereas for small *d *taxa counting grows linearly with sample size, it saturates clearly for *d ≥ 7 *at large sample sizes. Similar behavior was obtained for human 6 (not shown).

#### Sequence Error and count analysis

The raw data contain errors, and every misidentification of an amino acid will affect our taxa counts. Error rates can be estimated directly from the quality scores of the raw short reads. Their median indicates about 0.25 base errors for a short read of 72 bases, i.e. the probability for having an error in a single nucleotide is less than 0.35%. Since some of the nucleotide errors may leave the resulting amino-acids intact, we may estimate 1% to be an upper bound on the probability of an error in an amino acid designation within any one of the PPs.

To demonstrate the effect of such errors we study artificial sets of short reads by injecting arbitrary mutations into the original PP sets at the rate of 1%. The results are demonstrated in Figure 6C for raw short reads that we have already classified as belonging to SYV genes. We find that the *d ≥ 2 *count of the set with artificial errors (dashed curve) lies close to the *d ≥ 1 *curve of the original analysis (solid curve). In other words, the *d ≥ 2 *curve is a true estimate of a *d ≥ 1 *count on a problem without artificial errors. This allows us to conclude that the original *d ≥ 2 *count may be trusted to account for elimination of the majority of the true errors that exist in the original *d ≥ 1 *estimate. Clearly the errors infiltrate all other *d *counts, however they diminish considerably as *d *increases. We conclude that counts of 200 or more for sample sizes of order 1000, which are based on distances of d ≥ 2 and d ≥ 3, should already be regarded as correct. The fact that they keep increasing with sample size indicates that increasing depth unravels increasing numbers of strains and species.

Another interesting aspect of Figure 6 is that for *d ≥ 7 *the taxa counts saturate at about 60, providing a stable bound on the number of species that are expected to have quite large Hamming distances (over 150) between their relevant protein sequences. Similar behavior was obtained for human 6 (not shown).

#### Taxonomic identification - raw short reads

**Figure 7:**
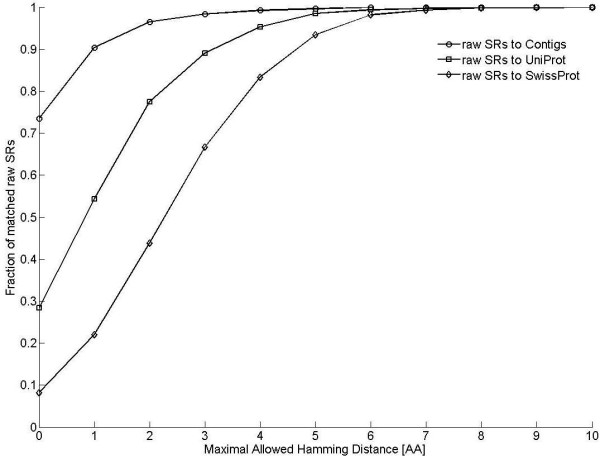
**Matching of raw short reads to fused contigs and Uniprot databases**. All short reads of length 24 amino-acids containing the leading SP of 6.1.1.9 were matched to the set of all 1009 fused contigs as well as to the enzyme data bases of Swiss-Prot and Uniprot. Values are presented for different numbers of allowed mismatches.

An interesting remaining question is whether we can identify the taxa from the raw short reads that we have analyzed. In Figure 7 we search for the proximity of all human 12 raw short reads, containing the leading SP of 6.1.1.9, to three data sets: All Swiss-Prot entries, all Uniprot entries, and our set of fused contigs corresponding to the same leading SP. We conclude from this figure, using the information at Hamming distance 1, that human 12 raw short reads contain about 10% novelties with respect to the set of contigs. Moreover, there are about 45% novelties when compared to all Uniprot enzymes. It should be kept in mind that all these short reads of length 24 amino acids have the same SP (of length 9) in common.

In Uniprot there are 96 Bacteroidetes proteins (74 contain the leading SP) and 611 Firmicutes (578 contain the leading SP) belonging to SYV. For the short reads matched to Uniprot, we have found 62 Bacteroidetes and 102 Firmicutes. Estimating the abundance of these principal phyla, by comparing with an unbiased set of proteins from different phyla, we conclude that Bacteroidetes is the major phylum. The next leading phyla are Firmicutes, Proteobacteria, Spirochaetes and Chloroflexi. To further support the significance of this result, we have repeated the same analysis comparing artificial short reads of length 24 amino-acids to the full Uniprot proteins from which they were drawn (Additional file 1, Fig. S5). Good matches (i.e., of hamming distance 0 or 1) between different phyla are very small. Thus, the large fractions observed in Figure 7 are indicative of true matches of the short reads to the quoted phyla.

## Discussion

### Use of single-copy genes

The idea that single-copy proteins can be used for estimating the number of species in metagenomes is well-known. In the first Sargasso-Sea metagenomic study, Venter et al. [17] have used the proteins AtpD, GyrB, Hap70, RecA, RpoB, and TufA to carry out such estimates. One of them, RpoB, is the basis of the analysis proposed by [4]. GyrB, has been used in an SP study of the Sargasso Sea data [14], leading to an estimate close to the one by Venter et al. [17]. Here we propose a method for taxa-counting in metagenomic data that is based on another type of single-copy genes - enzymes which belong to the aminoacyl tRNA synthetases super-family. Some of the aaRS enzymes are included in the 40 protein-coding marker genes of MLTreeMap [18], a method which sorts out the mapping of a species along the Tree of Life. Notably they include SYV, but exclude SYQ and SYT that play important roles in our analysis of the gut microbiome.

### The methodology, SPs search on aaRS enzymes

There are three important building-blocks in our proposed methodology. The first is relying on aaRS enzymes that have the characteristics of single proteins (the S61 set). The second is employing a look-up table of Specific Peptides (SPs) which serve as markers that, when encountered on metagenomic sequences, provide evidence for the association of these sequences with single-gene enzymes. The third is using the largest sets of sequences that are associated with single SPs as the basis for our taxa-counting algorithm. This is an NP-hard problem. Nonetheless, the fact that we deal with sets of sequences renders it solvable, even for thousands of such strings. In practice, we find that a large number of such sequences are inconsistent with one another (in the sense explained in the paper) and can be counted and removed from further analysis in the first step. Many of those that are left fit into sets within which sequences are mutually consistent with one another.

In the Additional file 1 we provide a link to the list of SPs that we have used in this study. A Matlab code implementing our counting algorithm is provided online. This should make it easy for metagenomic research groups to employ the tool that we propose, and establish its usefulness in future analyses.

### Application to long contigs

When analyzing long contigs it seems only natural to compare our methodology to the conventional 16S rRNA one. Working with an artificial metagenome composed of 64 genomes we have demonstrated that we have comparable achievements. We have employed the contigs of Qin et al. [10] to demonstrate how our proposed technology can work on real metagenomic data. We have separately applied it to their set of prevalent genes, and to their total data derived from gut microbiota of 124 individuals. Concentrating on estimates based on SYV genes (EC = 6.1.1.9) we find overall numbers of all the relevant contigs to be 912 in the prevalent gene set. However, we have estimated that a strict lower limit on the number of species should be 463, which was consistent with our estimate based on SYT genes (EC = 6.1.1.3). This estimate is a conservative number, making sure that no double counting error is made due to the presence of small factions of genes. Confronting these results with the statement of Qin et al. [10] about the existence of 1000 to 1150 prevalent species, we find that their result reflects the maximal number sustained by the data; however a cautious estimate of the lower limit reduces it by a factor of two.

Using the concept of prevalent genes one circumvents the issue of sorting out different strains from different species. We have compared these results to the results obtained by applying our method directly to the data of all 124 individuals. There is a fundamental difficulty in defining a threshold that separates different strains of the same species from different species, which is also well-known in the 16S rRNA analysis. It is also known to vary among different genera [19]. Within our method it is seen to vary among different ECs. This is exemplified in our analysis by comparing Figure 4 and 5: for SYV genes within Uniprot bacteria, strains of the same species exhibit sequence differences up to a distance of 10 amino-acids (Figure 4A). Different species demonstrate also such small differences in SYV sequences (Figure 4B), therefore strains and species counts cannot be separated by a distance criterion. Note that prevalent SYV genes have distances larger than 10 amino-acids (Figure 5A). Our SYV-based count of the total strains + species on all contigs is 1009 for the leading SP hits and 1136 after adding all non-leading SP strings that are long enough to avoid double-counting. This estimate is subject to an overall error of about 200 due to errors in contig construction (following the estimates of [10]). The separation between species and strains can however be carried out for SYQ genes: the bulk of strains in Uniprot data exhibit sequence differences below a distance of 3 amino-acids (Figure 4D) within the same species. In the SYQ-based count one can reach a lower bound estimate of different strains if one takes seriously observed differences of one or two amino-acids in the fused sequences generated from the data (Figure 5B). This particular family of enzymes, which carries rich information in the data of [10], was inapplicable to the problem of the artificial metagenome considered before in Table 3. The reason is the low coverage of SYQ genes in Swiss-Prot data (see the analysis of all bacteria in Additional file 1, Fig. S2).

### Taxa-counting independently of taxonomic assignment

The large diversity of bacterial life poses quite a challenge to the definition of what a "species" is. It has been suggested that sequence similarity is not always indicative of true diversity [20]. Nevertheless, advances in our understanding of single-gene properties may provide improved methods for estimating taxonomic diversity and composition [21,22].

Although our method is based on sequence similarity as well, it is different in various respects. Our taxa-counting algorithm does not go through a taxonomic assignment stage in order to arrive at its estimate of the count. In fact it relies on SPs that are common to all taxa, and counts the number of independent sequences, all of which contain the same SP. The fact that no comparisons to known data-bases (other than the list of SPs) are being used has a conceptual advantage: new species may belong to novel families and orders that have not been annotated so far. Comparing proteins from such species with known data bases may point out similarities to known species; but, unless the latter is based on extremely high amino acid identity, the results could be false (see Figure 1). It should be emphasized that even if new aaRS enzymes have low homology to those of known species, we may detect them as long as they carry one of the 4000 SPs on our look-up table.

Clearly, when long reads (such as Sanger-based methods) are being used, or long contigs, one can rely on BLAST comparisons of the extracted putative proteins with well annotated databases, to carry out the taxonomical classification of the data, and attempt to resolve the taxa-counting problem. This is the basis of many proposed algorithms [18,23,24], including the recently updated MEGAN algorithm [25,26]. Moreover, we can perform quite well using our own TSP approach [8] that resolved half of all fused strings of 6.1.1.9 into Bacterioidetes, Firmicutes and Proteobacteria.

### Phylogenetic tree

Phylogenetic trees are well-accepted tools in metagenomic studies. They may be based either on distance patterns inferred from grouping of 16S rRNA OTUs [27], or on similarities of protein sequences [18]. Using a single gene may be risky for inferring species phylogeny. In particular, it has been emphasized [6] that different aaRS enzymes may lead to slightly different evolutionary patterns, indicating also the existence of horizontal gene transfer among bacteria. Although we find that an analysis of all bacterial 6.1.1.9 data in Swiss-Prot leads to reasonable groupings of the relevant species, we know that such an analysis cannot be applied directly to the 1009 fused strings of 6.1.1.9. The reason is that the set of all contigs has a large fraction of short strings together with full proteins.

### Application of taxa counting to short read data

In the realm of short reads our method is quite unique. Having based it on SPs whose length is 9 or more amino-acids, we trust that the short reads that were selected by SP hits are fractions of the relevant enzymes. This allows for a quick species count estimate directly from raw data. Different strains cannot be distinguished from one another on the basis of such short reads. Short read singletons that are often discarded from metagenomic analysis because they do not combine with other short reads to form longer contigs, can be included in our analysis. By allowing for different minimal Hamming distances we can sort out such data, eliminate errors and provide interesting insights.

Our analysis suggests that counts based on raw short read data increase linearly with the sample size because of increased sequencing depth. This type of inflation has been observed as well in the number of OTUs [28]. When we focus our attention on large Hamming distances between the selected short reads, our counts saturate, indicating a lower bound of sufficiently distinct "species", presumably counts of different families.

### Taxonomic identification

Direct analysis of short reads has been proposed by [5], with their CARMA web-tool which is based on annotated environmental gene tags (EGTs). The latter may be as short as 27 amino-acids and were estimated to have specificity of 93% and sensitivity of 61% at the level of order prediction. Our proposed application is rooted in a different approach. Whereas both methods deal directly with short reads, we have aimed to achieve estimates for strict lower bounds of taxa counts, and the taxonomic identification is an interesting by-product.

An alternative method for taxonomic assignment using SPs has been proposed in [8] in terms of taxa-specific SPs (TSPs). This method cannot be applied to the restricted set of short-reads containing just the leading 6.1.1.9 SP. The reason is that the chances of establishing an additional TSP on a short read of 24 amino-acids, which includes already the leading SP, are nil. It can however be applied to the set of all raw short reads. We have carried out such an analysis (not displayed here) leading to results that are consistent with previous studies of the gut and with results obtained by matching raw-short reads, or contigs, that carry leading S61 SPs, to the Uniprot database.

## Conclusions

A major advantage of our methodology is its simplicity: its straightforward implementation does not require any further choice of parameters, or comparisons with additional data bases. The application to the microbiome data should serve as a validation of the power of our proposed methodology. The only bias in our methodology is the reliance on existing enzymes in Swiss-Prot, from which the SPs are being extracted. It should be noted that SPs belonging to the aaRS enzymes have the highest coverage among all SPs [8], because these enzymes permeate throughout all the tree of life. Hence they are expected to show up also in novel species. Nonetheless, it is theoretically possible that some genes of new species will be so far removed from the known ones that no SP match will occur and they may thus avoid detection. This danger should decrease with time. The reason is simply that as more and more data become available, the MEX algorithm [29] should be reapplied and it will pick up more motifs, thus increasing the set of S61 SPs and leading to higher recall.

The ever-improving sequencing technologies lead to rapid increases in the amounts of metagenomic data that become available. We believe that the method and concepts presented here can contribute significantly to such analyses in conjunction with other existing methods and techniques.

## Methods

### The Specific Peptides Approach

Kunik *et al. *[9] have extracted very short (~8aa) deterministic motifs, named Specific Peptides (SPs), whose presence in the protein sequence is a good marker for enzymatic functions by employing the motif-extraction technique MEX [29]. SPs are selected for their specificity to levels of the Enzyme Commission (EC) 4-level functional hierarchy.

Weingart *et al. *[14] have demonstrated how SPs can be employed for Data Mining of Enzymes (DME) on any given protein sequence. Their methodology relies on coverage length (L, overall number of amino-acids) of SP hits that carry the same EC assignments. In their analysis, they have chosen L ≥ 7. Increasing L improves precision and decreases recall of protein enzymatic annotations.

Weingart *et al. *[8] have applied the SP methodology directly to short reads, obtaining enzymatic and taxonomic signatures of the data. They have defined the S61 set and employed it for deriving taxonomic signatures of metagenomic data. Here we make use of all SPs belonging to S61 enzymes, but require L ≥ 9 in order to ensure better precision. Our aim is to determine a lower bound on the number of taxa (species families, genera, orders and classes, depending on the length of the reads) without specifying what they are.

### Estimating the specificity of SPs in a genomic study of *Escherichia coli*

In order to establish the expected error rate of EC annotation by SPs we perform an analysis of the *E. coli *genome that contains 4,639,675 nucleotides. We convert it in six possible ways to a long string of amino-acids and search for SP hits on them. We find 20,073 such records. SP hits in the genic regions are compared with known NCBI and Swiss-Prot EC annotations in the corresponding genes. They are then classified as true-positive (TP) or false-positive (FP) accordingly. SP hits on intergenic regions serve as a convenient 'negative set' to define random false-positive hits. We find only 60 hits on intergenic sections, with 53 of SP lengths L = 7, 6 of L = 8, and 1 of L = 10.

The total size of intergenic regions is 4,639,675 (Total Genome Length) -4,132,557 (Total CDS) = 507,118 nucleotides. From these data we estimate the number of FPs that could have been expected to occur in the genic regions. They are denoted as EFP (expected FPs) in Table 4, where we analyze the results of SP hits in the genic regions as function of the SP length, L. The errors of the genic regions are defined as FP/(FP+TP).

**Table 4:** Analysis of SP hits on the *E.coli *genome.

L	FP	TP	error	EFP
7	965	7,485	0.11	413

8	237	4,235	0.053	49

9	100	2,346	0.041	

10	68	1,361	0.048	8

Whereas for L = 7 the EFPs may account for a large fraction of the observed FPs, the former practically vanish for L ≥ 9. There are two important conclusions form this analysis. First, we conclude that L = 9 and 10 results indicate that 4-5% of the expert annotations may be wrong (which may also be due to modified EC classifications). The second is that using SPs of length L = 9 and 10 we should expect errors of less than 1%. Hence we limit our analysis in this paper to SPs of length L ≥ 9.

### Data set of Qin et al. [10]

We make use of the data of [10] who have analyzed gut metagenomics of 124 individuals, with two of the individuals (numbers 6 and 12) at a sampling depth larger than the others. The Illumina GA reads have been assembled into contigs for each individual, with estimated assembly errors of 14.2 per megabase. The authors obtained a sum total of contigs which they estimate to cover 14 M ORFs (longer than 100 bp each). From these they extracted a non-redundant set of 3.3 M ORFs that were termed 'prevalent genes'. This extraction removed redundant ORFs by pair-wise comparison, using a very stringent criterion of 95% identity over 90% of the shorter ORF length, which can fuse orthologues but avoids inflation of the data set due to possible sequencing errors (direct quote from [10]). The average length of the ORFs in the prevalent genes set is 704 bp.

The authors estimate that there exist between 1000 to 1150 prevalent bacterial species in the cohort of gut microbiota. This is based on the assumption that an average-sized bacterial genome contains about 3,364 genes, thus accounting for the 3.3 M ORFs in the prevalent set.

In our analysis we have made use of the contigs of both the prevalent genes, as well as the redundant set of all human data. The latter allows us to make contact with the former by searching for sequences with varying Hamming distances. Finally we have also made use of the raw data (Illumina GA reads) of individuals 6 and 12. These allow us to demonstrate that our method is applicable to data containing a large fraction of singletons that cannot be fused into contigs.

## Abbreviations

SP: specific peptides; PP: putative peptides; MEX: motif extraction algorithm; EC: enzyme commission classification; ORF: open reading frame; NW: Needleman Wunsch.

## Competing interests

The authors declare that they have no competing interests.

## Authors' Contributions

E. P. carried out the data analysis and developed the taxa counting algorithm. U. W. constructed the list of the S61 SPs and assisted in the data analysis. S. F. carried out the 16S rRNA analysis. D. H. conceived the research, participated in data analysis and wrote the paper with the assistance of E.P. All authors have read and approved the final manuscript.

## Supplementary Material

Additional file 1 **Supplementary material**. This file contains six sections. Section 1 contains a histogram of the lengths of SYV enzymes (Fig. S1). Section 2 is devoted to statistics of Uniprot KB data relevant to the three enzymes families used in this paper, and exemplified by the Venn diagram of Fig. S2. Section 3 is devoted to a demonstration of distances between *E coli *SYQ enzymes (Fig. S3). Section 4 discusses briefly Needleman Wunsch statistics of Uniprot KB (Fig. S4). Section 5 discusses match of short reads to proteins in Uniprot KB (Fig. S5). Section 6 provides links to a Matlab package for the taxa counting algorithm and to a C package for searching SP hits on PPs. The latter includes also a list of all SPs used in this paper. Click here for file
