# Supplementary material for: Peptide markers of aminoacyl tRNA synthetases facilitate taxa counting in metagenomic data
Source: BMC Genomics. 2012 Feb 10;13:65. doi: 10.1186/1471-2164-13-65 (PMC3319421; doi:10.1186/1471-2164-13-65)
Supplement: Additional file 1 — Supplementary material. This file contains six sections. Section 1 contains a histogram of the lengths of SYV enzymes (Fig. S1). Section 2 is devoted to statistics of Uniprot KB data relevant to the three enzymes families used in this paper, and exemplified by the Venn diagram of Fig. S2. Section 3 is devoted to a demonstration of distances between E coli SYQ enzymes (Fig. S3). Section 4 discusses briefly Needleman Wunsch statistics of Uniprot KB (Fig. S4). Section 5 discusses match of short reads to proteins in Uniprot KB (Fig. S5). Section 6 provides links to a Matlab package for the taxa counting algorithm and to a C package for searching SP hits on PPs. The latter includes also a list of all SPs used in this paper. [file 1471-2164-13-65-S1.DOC]

**Additional file 1 - Supplementary material**

This file contains six sections. Section 1 contains a histogram of the lengths of SYV enzymes (Fig. S1). Section 2 is devoted to statistics of Uniprot KB data relevant to the three enzymes families used in this paper, and exemplified by the Venn diagram of Fig. S2. Section 3 is devoted to a demonstration of distances between *E coli* SYQ enzymes (Fig. S3). Section 4 discusses briefly Needleman Wunsch statistics of Uniprot KB (Fig. S4). Section 5 discuss match of SR to proteins in Uniprot KB (Fig. S5). Section 6 provides links to a Matlab package for the taxa counting algorithm and to a C package for searching SP hits on PPs. The latter includes also a list of all SPs used in this paper.

1. **Lengths of SYV enzymes**.

In our lower bound estimate of species number we make use of the fact that full enzymes of the SYV type have a length of about 900 amino-acids. This is based on known Swiss-Prot data. It can also be corroborated by a meticulous examination of the data of Qin et al. (2010). Using the analysis of all contigs that possess the leading SP, and limiting ourselves to those contigs on which we observe both the start and stop signals we obtain the length distribution displayed in Fig. S1. Out of the total of 371 sequences, there exist 21 whose length is smaller than 700 amino-acids, which is the cut employed by us in the analysis. Thus we estimate that there exist 6% of short enzymes among all SYVs in the data. The effect of such an error on our c lower level estimates is minute: in the prevalent set we have counted 409 sequences for the leading SP, which was then amended by 54 additional ones from non-leading SPs. In the latter we have used the length limitation. This could have led to missing 3 candidates in the overall noise set of short sequences that we have neglected.


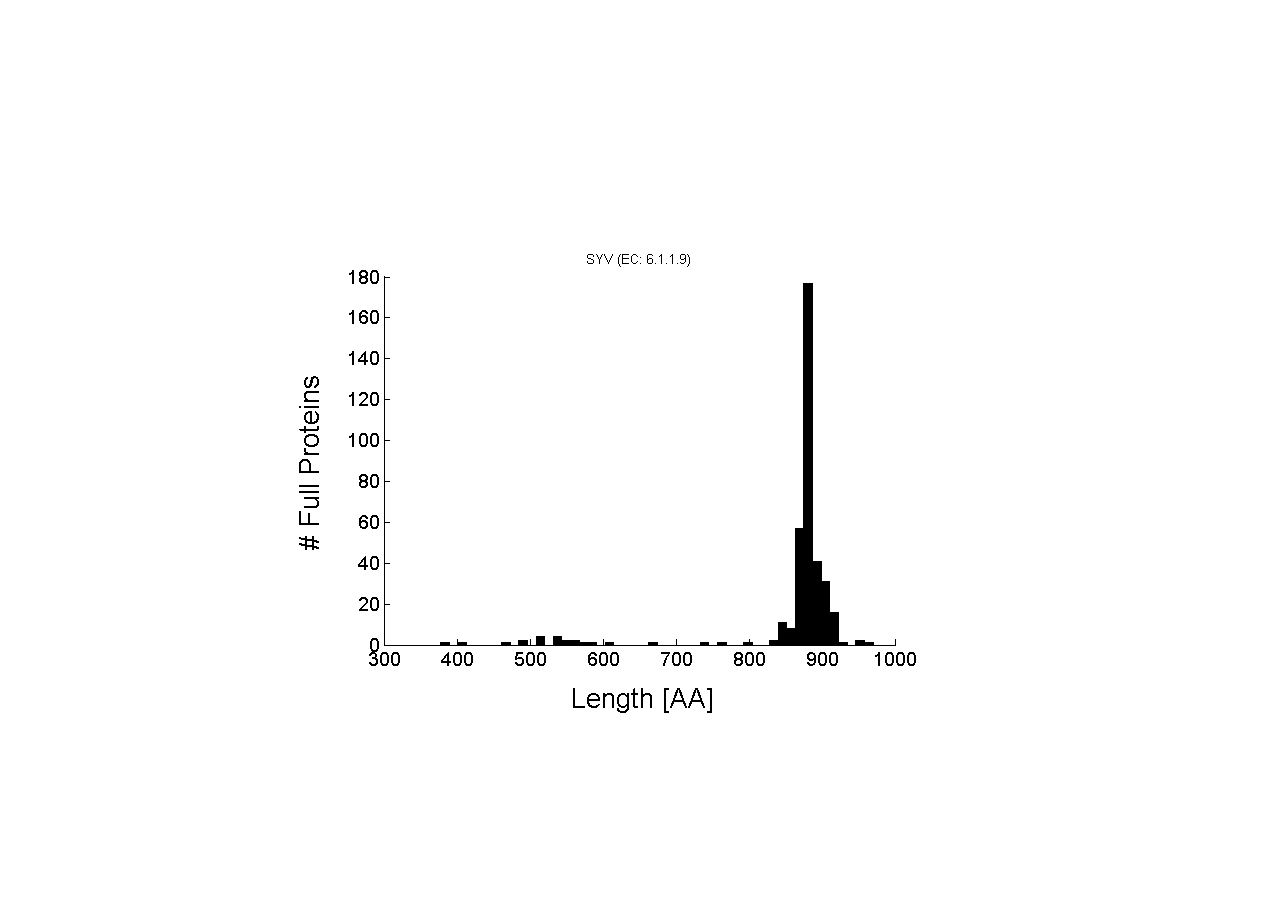


Figure S1. The histogram of length distribution of full enzymes (translated from start to stop) of EC= 6.1.1.9, carrying the leading SP.

1. **Statistics of Uniprot KB data.**

We have analyzed the sequences of all bacteria in Uniprot KB (including both Swiss-Prot and Trembl) in order to extract information on expected differences in aaRS sequences. There exist 2374 distinct species and strains. We have concentrated on ECs 6.1.1.3, 6.1.1.9 and 6.1.1.18, corresponding to the leading SP hits discussed in our metagenomic analysis. The distribution of the occurrence of such enzymes in the total data is displayed in the Venn diagram of Figure S2. There exists a large overlap between 6.1.1.3 and 9, both of which cover most of the data. In contradistinction, 6.1.1.18 enzymes are recorded only on about a third of all species (and their strains).

6.1.1.3

5

139

87

734

1151

22

21

None

6.1.1.18

6.1.1.9

Figure S2: Venn diagram of all bacteria in uniprot according to which ECs of 6.1.1.3, 6.1.1.9 and 6.1.1.18 exist in their records.

1. **Distances between E coli SYQ enzymes.**

The statistics of distance distributions for 6.1.1.18 are presented in Figure 3. Their details differ from the 6.1.1.9 ones presented there. In particular, there exists a much cleaner separation between distances of pairs of strains compared to pairs of different species. A threshold of distance < 3 seems to include most of all pairs of strains and exclude most of all pairs of species in 6.1.1.18. To exemplify this situation we present in Fig. S3 a dendrogram of multiple sequence alignment of the SYQ protein (6.1.1.18) on 21 strains of *Escherichia coli*, and one different organism within the same genus, *Escherichia fergusonii,* represented here as number 22. Here all distances are either zero or one. As a side remark note that, although *E. coli* is present in the microbiome data, it belongs to the minority of Proteobacteria, whereas most species are either Firmicutes or Bacterioidetes.

Figure S3. Dendrogram of multiple sequence alignment of SYQ proteins from 21 strains of *E coli* and one *E. fergussonii*.

1. **Needleman Wunsch Distance Matrix**

For Hamming distances that are well over 100 it is advisable to turn to edit distances. The latter take into account in an appropriate manner insertions or deletions that may account for very large Hamming distances. We make use of the Needleman-Wunsch (NW) edit distance [ref]. In Fig. S4a we present an analysis of Uniprot data of 6.1.1.9 and 6.1.1.18 NW distances of pairs of strains in species, species pairs in the same genus, and enzyme pairs of different genera belonging to the same family. Whereas the categories of strains and species look very similar to the Hamming distance analysis of Fig. 4 in the main text, we see differences in genera distributions between the NW analysis and the Hamming analysis, as expected.

Fig. S4b provides an insight into the different distributions of pairs of enzymes from different families, orders and classes. The similarity of these distributions does not allow us to formulate criteria for clear separation between these categories on the basis of NW distances. The statistics of different classes of 6.1.1.18 is very meager, because the data are dominated by essentially one phylum, proteobacteria, with a very little admixture of firmicutes belonging to just one class, clostridia.

The NW distance takes into account the effect of insertions and deletions. Hence it presents a better evolutionary picture than the Hamming distance, when homologies of proteins from different families are involved. However, the NW distances suffer from the inability to compare proteins (or sections of proteins) which have considerably different lengths, whereas the Hamming distance is always geared to adjust to the shorter sequences. Hence it is necessary to use the latter in our discussions of fused strings. In the analysis of full proteins belonging to different strains of the same species, or different species of the same genus, the two methods provide similar estimates (compare fig S4a a-b with fig 4 a-b).


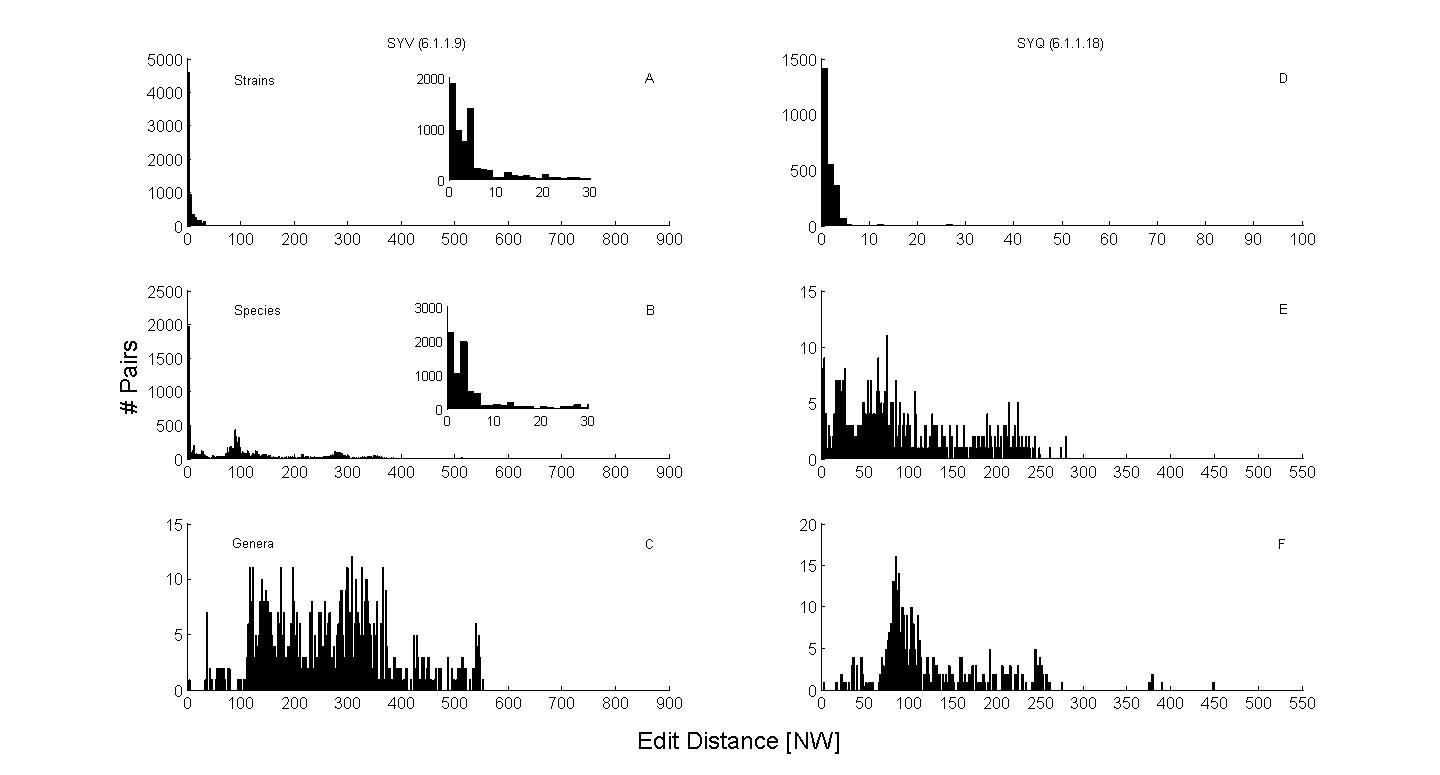


Figure S4a. Statistics of NW edit distances between 6.1.1.9 (A-C) sequences and 6.1.1.18 sequences (D-F) in Uniprot KB Data. Top: Differences between strains of the same species (A,D). Middle: Differences between species of the same genus (B,E). Bottom: differences between genera in the same family (C,F). Comparison with Fig. 4 in the main text shows that NW edit distances are significantly different from Hamming distances for the cases C and F.


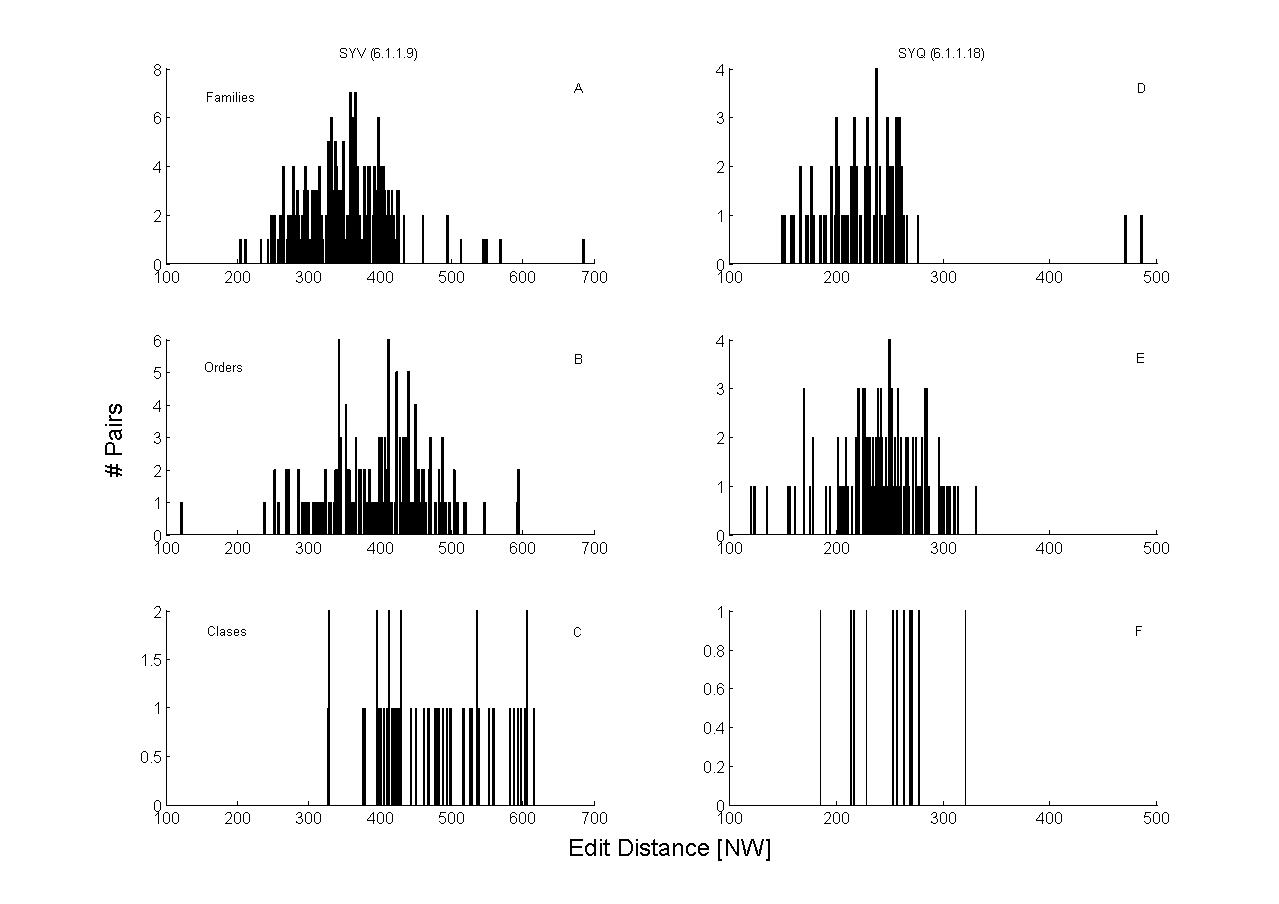


Figure S4b. Statistics of NW edit distances between 6.1.1.9 (A-C) sequences and 6.1.1.18 sequences (D-F) in Uniprot KB Data. Top: Differences between families of the same order (A,D). Middle: Differences between orders of the same class (B,E). Bottom: differences between classes in the same phylum (C,F).

1. **Match of artificial short reads to full proteins – Uniprot KB**

Short reads of length L = 24 amino-acids have been artificially constructed from the full set of SYV (6.1.1.9) proteins in Uniprot sharing the same SP. These short reads were then compared to the full proteins by SP alignment to produce figure S5. The very small fraction at hamming distances 0 and 1 indicate the significance of the results presented in figure 7.

**
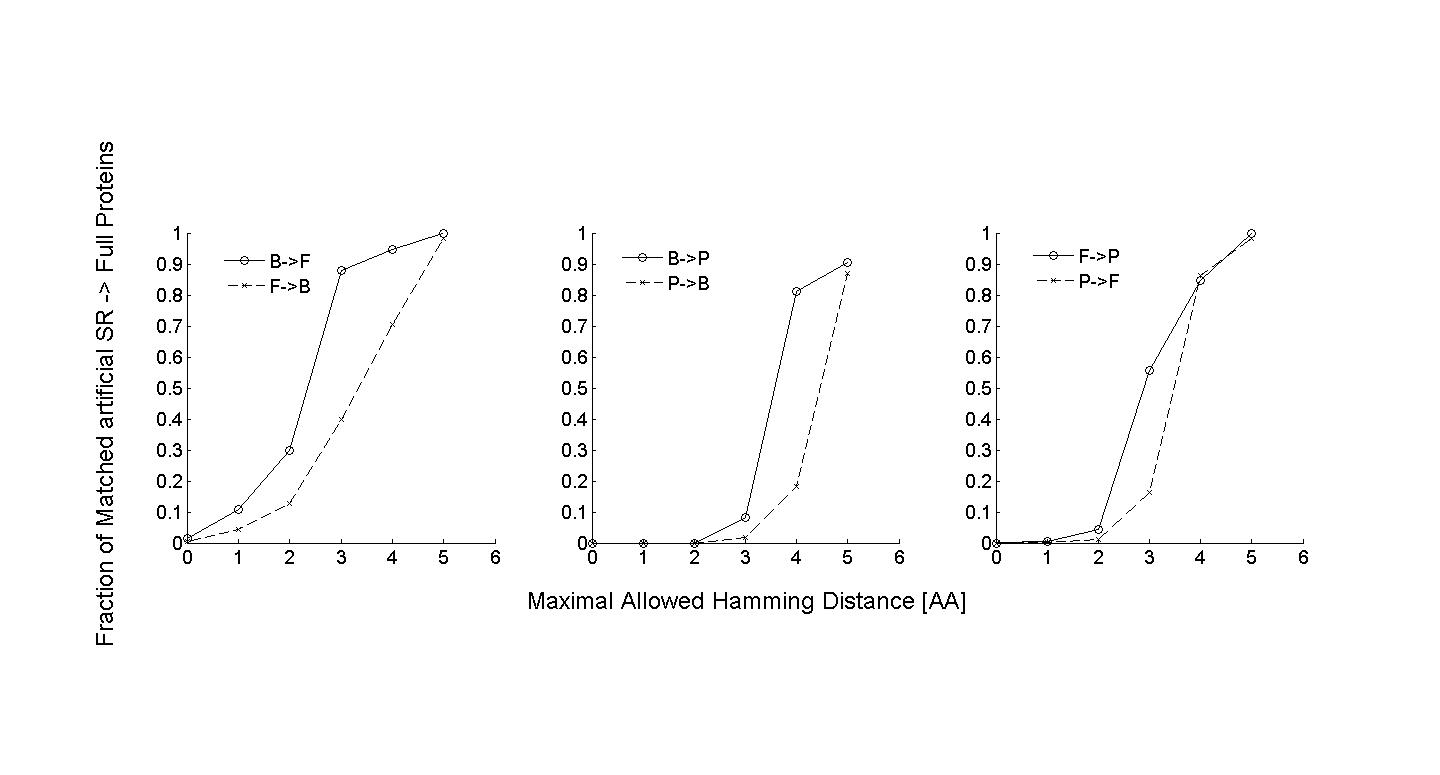
**

Figure S5. Statistics of Uniprot data for the principal phyla Bacteroidetes (B), Firmicutes (F) and Protobacteria (P). Curves show the faction of matched artificial raw short reads of length 24 amino-acids to full proteins from which they were drawn as function of the maximal allowed hamming distance between any two comparisons. Left) for B and F. Middle) For B and P. Right) For F and P.

1. **Software and SP lists**

We provide two links to facilitate the calculations needed to apply the method developed in this paper.

<http://horn.tau.ac.il/SC.html> contains a matlab code that performs the **species counting** algorithm (referred to as taxa counting throughout this paper) on a set of putative peptides that contain the same SP.

<http://horn.tau.ac.il/SCC.html> contains a C code that searches for **SP hits** on a list of putative peptides. Here we provide also a list of all 3949 SPs that we use (SP_S61.xls) and their corresponding EC numbers (ECs_S61.txt).
